# Propagation and Molecular Characterization of Bioreactor Adapted Very Virulent Infectious Bursal Disease Virus Isolates of Malaysia

**DOI:** 10.1155/2018/1068758

**Published:** 2018-09-02

**Authors:** Nafi'u Lawal, Mohd Hair-Bejo, Siti Suri Arshad, Abdul Rahman Omar, Aini Ideris

**Affiliations:** ^1^Department of Veterinary Pathology and Microbiology, Faculty of Veterinary Medicine, Universiti Putra Malaysia (UPM), 43400 Serdang, Selangor, Malaysia; ^2^Department of Veterinary Microbiology, Faculty of Veterinary Medicine, Usmanu Danfodiyo University, Sokoto (UDUS), 2346 Sokoto, Nigeria; ^3^Laboratory of Vaccine and Immunotherapeutics, Institute of Bioscience, Universiti Putra Malaysia (UPM), 43400 Serdang, Selangor, Malaysia; ^4^Department of Veterinary Clinical Studies, Faculty of Veterinary Medicine, Universiti Putra Malaysia (UPM), 43400 Serdang, Selangor, Malaysia

## Abstract

Two Malaysian very virulent infectious bursal disease virus (vvIBDV) strains UPM0081 (also known as B00/81) and UPM190 (also known as UPM04/190) isolated from local IBD outbreaks in 2000 and 2004, respectively, were separately passaged for 12 consecutive times in 11-day-old specific pathogen free (SPF) chicken embryonated eggs (CEE) via the chorioallantoic membrane (CAM) route. The CEE passage 8 (EP8) isolates were passaged once in BGM-70 cell line yielding UPM0081EP8BGMP1 and UPM190EP8BGMP1, while the EP12 isolates were passaged 15 times in BGM-70 cell line yielding UPM0081EP12BGMP15 and UPM190EP12BGMP15 using T25 tissue culture flask. These isolates were all propagated once in bioreactor using cytodex 1 as microcarrier at 3 g per liter (3 g/L) yielding UPM0081EP8BGMP1BP1, UPM190EP8BGMP1BP1, UPM0081EP12BGMP15BP1, and UPM190EP12BGMP15BP1 isolates. The viruses were harvested at 3 days after inoculation, following the appearance of cytopathic effects (CPE) characterized by detachment from the microcarrier using standard protocol and filtered using 0.2 *μ*m syringe filter. The filtrates were positive for IBDV by RT-PCR and immunofluorescence. Sequence and phylogenetic tree analysis indicated that the isolates were of the vvIBDV strains and were not different from the flask propagated parental viruses.

## 1. Introduction

Traditionally, infectious bursal disease virus (IBDV), the causative agent of infectious bursal disease (IBD), was isolated through inoculation of suspected samples into 9 to 11-day-old CEE via CAM route [1]. However, this technique is not always effective, because some variant strains of the virus do not induce embryonic mortality [2], an infection index that CAM inoculation methods mostly depend on to indicate productive infection. Advances in IBD diagnosis made it possible for many IBD virus (IBDV) isolates to be adapted on primary avian cell cultures derived from chicken embryonic tissues from organs such as kidney (CEK), bursa (CEB), and muscles (CEF) [3, 4]. These cells, however, were found to produce low virus titer, have limited cell growth, and may be a source of contamination with extraneous avian viruses [5, 6]. To address these issues research in cell culture technology leads to the discovery of cell lines of mammalian origins that are easy to handle and manipulate, have infinite lifespan, and are free from extraneous avian viruses. Many cells derived from mammals are widely in use now for the production of recombinant proteins such as enzymes, hormones, chemokines, cytokines, antibodies, and vaccines for therapeutic use in humans and animals, triggering the huge invested efforts in research and development of animal cells as production vehicles for commercialization of products [7]. Because of the soaring demand for safe cell culture products especially vaccines, there is an urgent need to increase the productivity of cells with minimal investments in resources/equipment and decrease microbial contamination and absolute control of production parameters, a need that has been met by the introduction of bioreactor technology [7, 8]. Infectious bursal disease vaccines are mainly produced using specific pathogen free (SPF) CEE which is costly and requires large facilities, difficult to scale up, laborious, and hindered by limited supply of SPF eggs [6]. In the case of vvIBDV, the limitations are even more due to the difficulty with which these strains adapt to cell culture [6, 9]. A paradigm shift to cell culture-based IBD vaccine using bioreactor technology will help in reducing the threat IBD posed to the poultry industry worldwide through rapid response in vaccine development, with a flexibility that would significantly reduce the time frame to develop and approve vaccines, thereby effectively decreasing the time from laboratory to market (Meuwly* et al*., 2006) [10]. The development of table top bioreactors scaled down from industrial sizes to fit in the laboratory and function efficiently while occupying minimum bench space revolutionized the field of vaccinology in both humans and animals [11]. Several cell lines are in use in the development of cell culture-based vaccines among which there are Vero [12, 13], CHO [12, 14], RK-13 (Rinaldi* et al*., 1972), and BGM-70 cells [12, 14, 15]. Several cell lines were utilized for producing IBD vaccines in a cell culture-based system [16, 17]. The cell line BGM-70 derived from baby grivet monkey was reported to support the growth and rapidly attenuate classical, variant, and very virulent IBDV strains and with high viral titer [6, 15, 18]. The ability of this continuous cell line to yield higher viral titers is a valuable characteristic that makes growing viruses in them advantageous over primary cell cultures apart from convenience. Despite these advantages, the viral titers obtained from continuous cell lines using traditional flasks culture method are often inadequate to meet the excessive demand and production speed required for poultry vaccines production to curtail production losses attributable to disease. This shortcoming is further complicated by the frequent bacterial and fungal contamination encountered when handling cells during culture techniques [19]. BGM-70 cell line was selected for the propagation of local Malaysian IBDV isolates in a table top bioreactor to evaluate the ability to support the growth of the viruses in the bioreactor. This is due to the high titer obtained when the cell line was used to propagate the virus in a conventional flask culture, confirming the earlier reports of its potential [14, 18, 20]; Abdel-Alim et al., 2003; [15].

Although the use of bioreactor to propagate viruses has been established, the propagation of vvIBDV using bioreactor system is poorly reported. It was the hypothesis of this study that the BGM-70 adapted and propagated IBDV could be well adapted and propagated on a microcarrier using bioreactor system. Therefore, the objectives of this study were to propagate vvIBDV of Malaysian isolates, UPM0081 and UPM190 in BGM-70 cells using bioreactor, and molecularly characterized the isolates.

## 2. Materials and Methods

### 2.1. IBDV Isolates and BGM-70 Cells

The vvIBDV isolates maintained as CAM homogenates were isolated from local outbreaks of IBD in Malaysia in the years 2000 and 2004 and were designated UPM0081 (also known as B00/81, AY520910) and UPM190 (also known as UPM04/190, AY791998), respectively. These isolates were separately passaged for consecutive 12 times in 11-day-old specific pathogen free (SPF) chicken embryonated eggs (CEE) via the chorioallantoic membrane (CAM) route. The CEE passage 8 (UPM0081EP8, accession number KY411643, and UPM190EP8, accession number KY411645) of the isolates was passaged once in BGM-70 cell line (EP8BGMP1) giving rise to UPM0081EP8BGMP1 and UPM190EP8BGMP1, while the CEE passage 12 (UPM0081EP12-KY411641 and UPM190EP12) of the isolates was serially passaged in BGM-70 cell line for 15 times (BGMP1-15) giving rise to UPM0081BGMP15 (accession number KY418012) and UPM190EP12BGMP15 (accession number KY418010) using T25 tissue culture flask. The virus titers obtained from BGM-70 flask propagation of EP8 isolates at passage 1 were 1.0 × 10^3.8^ TCID_50_/mL and 1.0 × 10^4.4^ TCID_50_/mL for UPM0081EP8BGMP1 and UPM190EP8BGMP1, respectively, while the titers obtained from BGM-70 flask propagation of EP12 isolates at passage 15 were 10^9.2^ TCID_50_/mL and 1.0 × 10^9.5^ TCID_50_/mL for UPM0081BGMP15 and UPM190BGMP15, respectively, as determined using standard technique described previously (Reed & Muench, 1938). These respective isolates were utilized for one time propagation in bioreactor (BP1).

The BGM-70 cell line is an epithelial-like cell derived from baby grivet monkey kidney (ECACC cat no. 90092601) obtained from the European Collection of Authenticated Cell Cultures (ECACC, Porton Down, Salisbury, SP4 0JG, UK) and was maintained in minimum essential medium (MEM) supplemented with 10% fetal bovine serum and 5% CO_2_ at 37°C. Fifteen confluent 25 cm^2^ flasks were used to seed the cells on sterile cytodex 1 microcarriers.

### 2.2. Prebioreactor Cultures

T-25 flasks (Corning) were seeded with approximately 2.5 x10^4^ cells/cm^2^ in a prewarmed MEM supplemented with 10% fetal bovine serum (FBS). The seeded flasks were incubated in an incubator at 37°C with 5% CO_2_ condition. Daily observation of each flask was conducted at least twice using an inverted microscope (Olympus® Japan) for complete monolayer formation. Following confluence, the cells were washed with prewarmed Ca^2+^/Mg^2+^ free Dulbecco's PBS (*D1408 SIGMA*) and detached from the flask with StemPro®Accutase® (A11105-01 Gibco, USA) whose dissociation activity was not stopped until all the cells became detached from the flasks. The dissociated cells were then used to seed the bioreactors.

### 2.3. Preparation of Microcarrier

Cytodex 1 microcarriers (#17-0448-01, GE Healthcare, Uppsala Sweden) was used at a final concentration of 3 g/L according to the manufacturer's instructions. The microcarriers were hydrated in siliconized glass containers using Ca^2+^/Mg^2+^ free Dulbecco's PBS (*D1408 SIGMA*) (pH 7.4) for 4 to 6 hours. After the hydration, the microcarriers were rinsed three times with Ca^2+^/Mg^2+^ free PBS, followed by autoclaving for 15 min at 121°C [21] inside the bioreactor (Biosys Fermetec Resources, Selangor, Malaysia) and the microcarriers were rinsed with 200 mL of serum free MEM before cell seeding.

### 2.4. BGM-70 Cell Culture in Biosys Fermetec Bioreactor System

For the cultivation of BGM-70 cells in a bioreactor system, a 2 L stirred tank bioreactor (Biosys Fermetec Bioreactor System) was used. A 200 mL of MEM supplemented with 10% FBS was added to the microcarriers and allowed to stand for 30 minutes. This was then followed by the addition of BGM-70 cells suspended in 800 mL of growth media at cell density of 1.5 X 10^4^/mL to the microcarriers. The culture conditions were 37°C, 7.2 pH (CO_2_ and NaHCO_3_ controlled), 50% air saturation for dissolved oxygen (DO) [22, 23]. To obtain even attachment to the microcarriers, the method of Gouzgeng* et al*. [11] was adapted with initial continuous stirring at 14 rpm/5 minutes followed by an increase in speed at 25 rpm/5 minute after 24 hours was used as the stirring condition [11]. The cultures were performed in triplicate. Virus cultures were conducted without media change at multiplicity of infection (MOI) of 1 and the cultures were harvested at 72 hours after inoculation at 100% cytopathic effect (CPE). To harvest, the bioreactor propeller was switched to full speed to agitate the microcarriers and facilitate cell dislodgement. The microcarrier was allowed to settle down before being sieved with a sterile 70 *μ*m cell strainer and the virus filtrate suspension was freeze-thawed three times and centrifuged at 500* x* g for 20 minutes at 4°C. The supernatant was filtered using 0.22 *μ*m syringe filter (Millex®, Merck Millipore, Germany) and aliquot and stored at −80°C until required.

### 2.5. Cell Culture Sampling and Analysis

For routine cell sampling, 5 to 10 mL of cells was sampled using a sampling bottle fitted to the sampling port. A 10 mL syringe fitted with a 0.22 *μ*m syringe filter (Millex®, Merck) was used to create a negative pressure on the other side of the sampling port which draws the sample from the bioreactor to the sampling bottle. Samples were taken at 6 and 24 hours post seeding (ps). Sampled cells were counted with a hemocytometer using trypan blue exclusion to determine cell viability. To count the cells, 800 *μ*L of supernatant was removed and replaced with an equal volume of 0.4% trypan blue. The suspension was gently mixed and the cells were counted in a hemocytometer chamber using inverted microscope (Olympus, Japan) at 10*x* magnification. The cell viability was calculated according to the following equation: *C* = *AVx*2*x*10^4^, where *C* is the cell concentration,* AV* is the mean number of cells in the 4 chambers counted, and* 2* is the dilution factor. Samples were observed to determine attachment of the cells to microcarriers using inverted microscope (Olympus, Japan) at 20*x* magnification [21]. The bioreactor filtrates were titrated in SPF chicken embryos through 10-fold serial dilutions of the filtrates and inoculation onto the CAM as described by Rinaldi* et al*. [24] and Hitchner [1]. Titers obtained were expressed as the 50% embryo infective dose (EID_50_) per milliliter and were calculated using the method of Reed and Muench (1938).

### 2.6. RT-PCR of the Bioreactor Propagated vvIBDV

The IBDV genomic RNA from the filtrates were extracted using Trizol kit (Trizol®LS) for RT-PCR analysis as recommended by the manufacturer. All the reaction setup was carried out on ice unless otherwise stated. The RNA was denatured by addition of 9 *μ*L of RNA, 1 *μ*L dimethyl sulfoxide (DMSO), 1 *μ*L of reverse and forward primers, and 4 *μ*L of DEPC water and the mixture was heated in heating block for 5 minutes at 65°C before it was placed immediately on ice for 5 minutes. Reverse transcription was carried out at 42°C for 1 hour in a total volume of 20 *μ*L containing 9 *μ*L of denatured RNA, 4 *μ*L MgCl_2_, 5 units of AMV, 4 *μ*L of reaction buffer, 2 *μ*L of dNTP, and 0.5 *μ*L Rnasin. After the reverse transcription, the reaction was stopped by heating the mixture to 99°C for 1 minute; thereafter, 5 *μ*L of cDNA was added to PCR reaction. This mixture contained 1 *μ*L of 10 mm dNTP, 4 *μ*L of MgCl_2_, 6 *μ*L of PCR buffer, 1 *μ*L of reverse and forward primers, 5 *μ*L cDNA, 31.5 *μ*L of water, and 0.5 *μ*L of Taq polymerase. The PCR reaction was performed in a thermocycler (SensoQuest, Germany) using the following temperature profile for 35 cycles: 95°C for 1 minute, 52°C for 1 minute, 72°C for 2 minutes, and lastly, the final extension was at 72°C for 10 minutes. The PCR product was analyzed by using 1% agarose gel in a Mini-Sub cell GT gel electrophoresis apparatus (Bio-Rad, USA). One Kb DNA ladder (Fermenters, USA) was used as marker by mixing 5 *μ*L of the ladder and 2 *μ*L of 6x gel loading dye (R0611, ThermoFisher). Other wells were loaded with 5 *μ*L of the RT-PCR products that were also mixed with 2 *μ*L of loading dye. The gel electrophoresis was run at 75 V for 40 minutes. At the end of the electrophoresis, the gel was stained with RedSafe nucleic acid staining solution (21141, iNtRON Biotechnology) for 30 minutes. The gel was placed onto UV illuminator to visualize the nucleic acids bands using gel documentation system (BioRad, USA).

### 2.7. Sequence Analysis

The PCR products were sequenced (First BASE Laboratories Sdn Bhd) and the identity of the sequence was confirmed using the basic BLAST (Basic Local Alignment Search Tool) search programme of National Centre for Biotechnology Information (NCBI) (http://www.ncbi.nlm.nih.gov). Sequence editing, multiple alignments, and analysis was done using MEGA version 7.0.4 [25, 26] with the parent BGM-70 and SPF eggs propagated UPM0081 and UPM190 isolates alongside other downloaded reference sequences to determine the changes in nucleotide and amino acid sequences that may be caused due to differences in propagation system.

### 2.8. Indirect Immunofluorescence Test

Indirect immunofluorescence (IIF) test was conducted to demonstrate the presence of IBDV antigen within the cytoplasm of infected BGM-70 cells inoculated with the bioreactor and tissue culture flask propagated UPM0081 and UPM190 isolates as described previously [15]. Briefly, clean cover slips were placed in 6 well tissue culture plates and allowed to stand overnight under UV light exposure. The plates were seeded with BGM-70 cells following standard established protocols [27]. Following confluence, the plates were washed twice with prewarmed PBS, and the individual wells were inoculated with 200 *μ*L of the harvested bioreactor and tissue culture flask supernatants of the UPM0081 and UPM190 isolates and incubated for 120 minutes for adsorption at 37°C and 5% CO_2_ condition, and then 1.8 mL of maintenance medium was added to each well, while the uninfected control wells were filled with 2 mL of the medium. The plates were then incubated at 37°C and 5% CO_2_ conditions for 3 days. The plates were fixed with 4% paraformaldehyde for 30 minutes at room temperature and washed three times for 5 minutes using ice cold PBST (PBS containing 0.5% Tween 20). After this, the plates were incubated in a 0.5% Triton X-100 in PBST (PBS 1L pH 7.2 + 0.5 mL Tween 20) for 15 minutes to permeabilize the cells followed by rinsing with PBST three times for 5 minutes. Unspecific binding was blocked using blocking buffer (5% BSA in PBST) for 1 hour at room temperature. The plates were rinsed three times with PBST for 5 minutes and then 40 *μ*L of chicken monoclonal anti-VP2-IBDV specific primary antibody (Charles River Laboratories, USA) diluted at 1:200 with sterile distilled water was dropped on the cells followed by incubation at 4°C in a dark humidified chamber overnight. The plates were washed thrice for 5 minutes with PBST and 40 *μ*L of polyclonal rabbit anti-chicken-FITC-conjugated secondary antibody raised against chicken IgY-Fc (SIGMA*-*ALDRICH, Germany) and diluted at 1:1000 with sterile distilled water was dropped on each slide containing wells and incubated at room temperature for 1 hour in the dark. The plates were rinsed with PBST for 5 minutes three times and 20 *μ*L of 4′,6-Diamidino-2-phenylindole dihydrochloride (DAPI) (SIGMA-ALDRICH, Germany) was added to the plates and incubated for 10 minutes at room temperature and the plates were briefly rinsed with PBST. The cover slips were dried and placed on a labeled clean glass slides using a mountant (DPX) for fluorescence microscopic examination (Leica Microsystems Limited, Heerbrugg, Switzerland) of VP2 antigen positive cells [28].

## 3. Results

### 3.1. BGM-70 Cell Culture in Biosys Fermetec Bioreactor System

**Figure 1:**
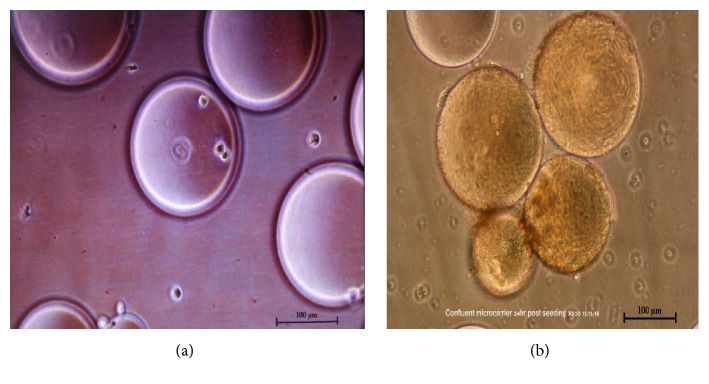
*BGM-70 cells attaching to cytodex microcarrier 1 in a bioreactor.* (a) BGM-70 cells attaching to cytodex at 6 hours ps. (b) Confluent cells on cytodex at 24 hours ps. Bar=100 *μ*m.

The BGM-70 cells started attaching to the microcarriers at 6 hours post seeding (ps) (Figure 1(a)) and became confluent 24 hours ps with cell density of 2.1 X 10^6^ cells/ mL (Figure 1(b)).

**Figure 2:**
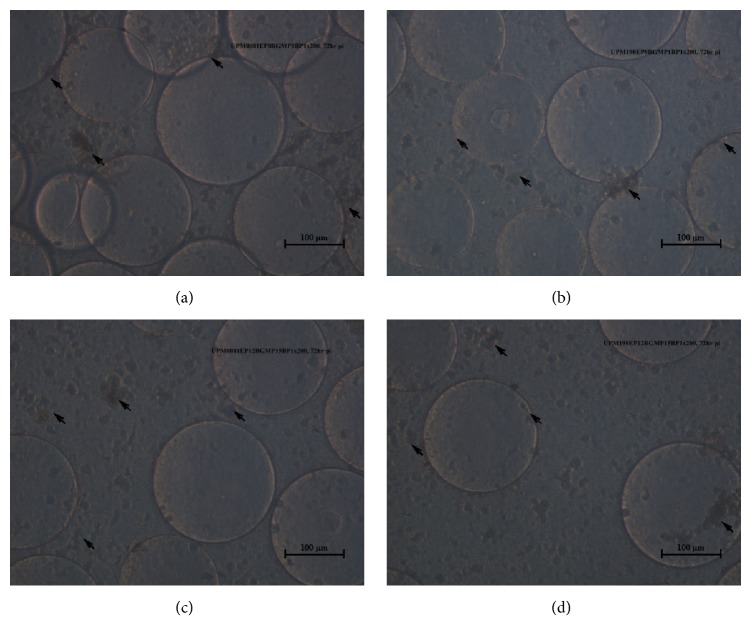
*vvIBDV infected BGM-70 cells detaching from cytodex.* (a) Detachment of BGM-70 cells from microcarriers at 72 hours pi with UPM0081EP8BGMP1BP1. (b) Detachment of BGM-70 cells from microcarriers at 72 hours pi with UPM190EP8BGMP1BP1. (c) Detachment of BGM-70 cells from microcarriers at 72 hours pi with UPM0081EP12BGMP15BP1. (d) Detachment of BGM-70 cells from microcarriers at 72 hours pi with UPM190EP12BGMP15BP1. Bar=100 *μ*m.

The CEE adapted UPM0081EP8BGMP1 passaged once in BGM-70 cells and propagated in the bioreactor also developed CPE between 48 and 72 hours that was characterized by cell detachment from the microcarrier. The propagated IBDV was harvested at 72 hours pi with a titer of 10^6.29^ TCID_50_/ 1 mL and was designated UPM0081EP8BGMP1BP1 (Figure 2(a)).

Similarly, the CEE adapted UPM190EP8BGMP1 passaged once in BGM-70 cells and propagated in the bioreactor developed CPE within 48 hours that was characterized by detachment from the cytodex microcarriers. At 72 hours pi, the virus was harvested with a titer of 10^6.4^ TCID_50_/ 1 mL and was designated UPM190EP8BGMP1BP1 (Figure 2(b)).

The confluent BGM-70 cells growing on microcarriers were infected with vvIBDV UPM0081BGMP15 with CPE development at 72 hours pi at which time, the virus was harvested. The CPE was characterized by cell detachment from the microcarriers. The harvested virus was designed UPM0081EP12BGMP15BP1 (Figure 2(c)) and the viral titer was 10^8.23^ TCID_50_/ 1 mL.

The confluent BGM-70 cells growing on the microcarrier were infected with vvIBDV UPM190BGMP15 with CPE development at 72 hours pi at which time, the virus was harvested and designated as UPM190EP12BGMP15BP1. The CPE was characterized by cell detachment from the microcarrier. The viral titer obtained was 10^8.24^ TCID_50_/ 1 mL UPM190EP12BGMP15BP1 (Figure 2(d)).

### 3.2. RT-PCR of the Bioreactor Propagated vvIBDV 

**Figure 3:**
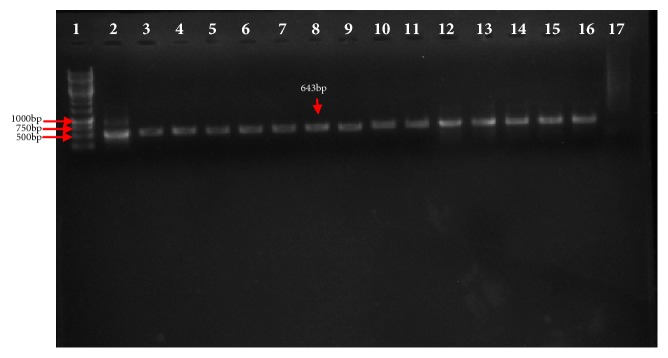
*Gel electrophoresis of bioreactor passaged viruses.* RT-PCR product showing 643 bp fragment of bioreactor passaged vvIBDV UPM0081EP12BGMP15BP1 (lane 2), UPM0081EP8BGMP1BP1 (lane 3), UPM190EP12BGMP15BP1 (lane 4), UMP190EP8BGMP1BP1 (lane 5), and original BGM-70 and egg passaged isolates UPM0081BGMP15 (lane 6), UPM190BGMP15 (lane 7), UPM0081EP8 (lane 8), UPM190EP8 (lane 9), UPM0081EP12 (lanes 10 and 11), UPM190EP12 (lanes 12 and 13), positive controls (lanes 14, 15, and 16), and negative control (lane 17). A 1000 bp DNA ladder (lanes 1 and 15) (MBI Fermentas, Lithuania) was used to flank the PCR products.

**Figure 4:**
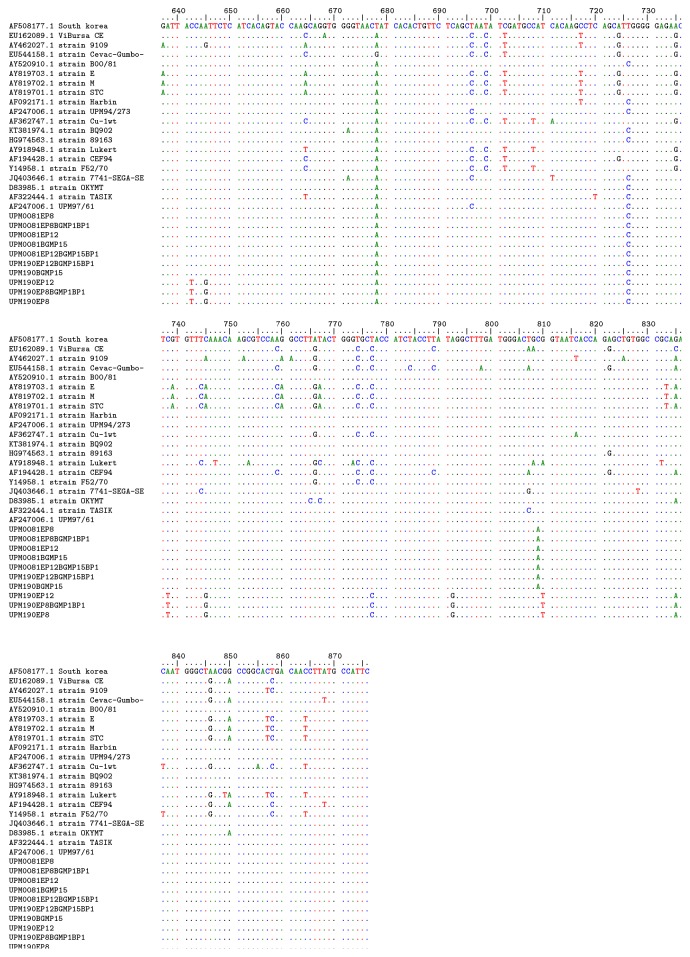
*Comparison of nucleotide sequences of bioreactor, conventional flask, and CAM propagated UPM0081 and UPM190.* Nucleotide alignment of bioreactor, BGM-70, and SPF egg UPM190 and UPM0081 isolates with 30 reference sequences showing no nucleotide changes between the original parent and the bioreactor passaged isolates.

**Figure 5:**
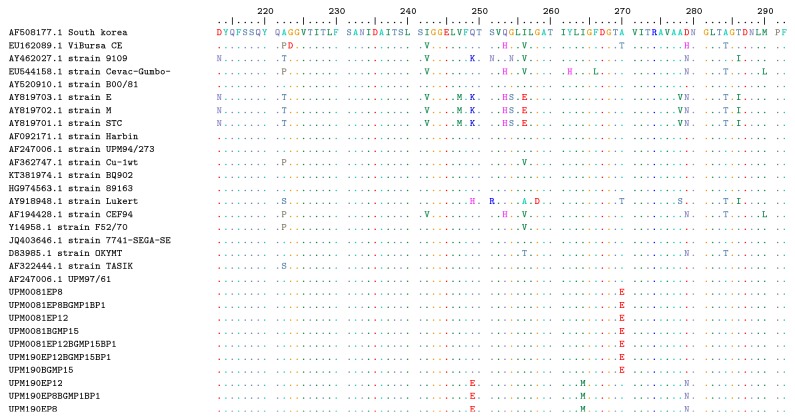
*Comparison of amino acid sequences of bioreactor and BGM-70 propagated UPM0081 and UPM190.* Protein alignment of bioreactor, BGM-70, and SPF egg passaged UPM190 and UPM0081 isolates with 30 reference sequences showing no amino acid changes between the original parental isolates and the bioreactor passaged isolates.

The PCR products upon electrophoresis revealed a 643 bp fragments (Figure 3) and sequencing results obtained were analyzed using MEGA version 7 [26] and Bioedit version 7 bioinformatic software [29] as shown below (Figures 4 and 5).

**Figure 6:**
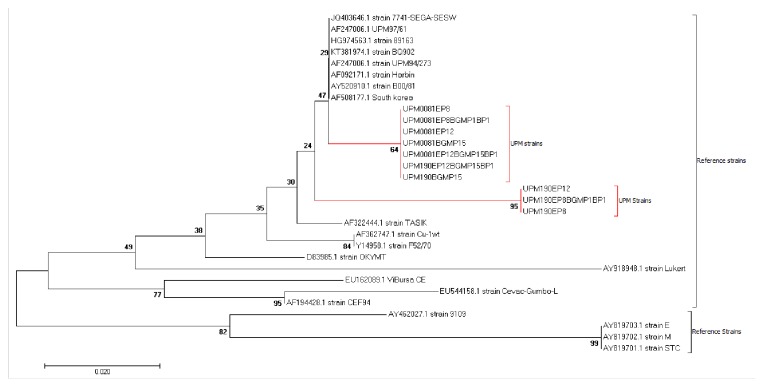
*Phylogenetic analysis of bioreactor, CAM adapted, and conventional flask propagated UPM0081 and UPM190.* Phylogenetic relationship of taxa between bioreactor, BGM-70, and CEE passaged UPM190 and UPM0081 isolates with reference sequences to determine their phylogenetic relationship. The evolutionary history was inferred using the Neighbor-Joining method with bootstrap (1000 replicates). The evolutionary distances were computed using Poisson correction method. The analysis involved 30 amino acid sequences with a total of 80 positions. The analyses were conducted in MEGA7. Note that the UPM strains formed 2 distinct clusters within the vvIBDV branches from the reference sequences indicating their close relationship.

Within the hypervariable region of VP2 from residue 213 to 292, at position 249, only UPM190EP8 and its bioreactor passaged isolates as well as UPM190EP12 had an E249, while the UPM190BGMP15, its bioreactor passaged isolate and all the UPM0081 BGM-70, egg, and bioreactor passaged isolates had Q249. Similarly, the UPM190EP8 and its bioreactor passaged isolates UPM190EP8BGMP1BP1 and UPM190EP12 had M264 against the I264 possessed by the rest of the viruses compared. At residue 270, the bioreactor passage and other viruses had E270 except the UPM190EP8BGMP1, UPM190EP8, and UPM190EP12 isolates that had A270 indicating the stability of this mutation in BGM-70 passaged viruses irrespective of the medium of propagation. The phylogenetic analysis revealed that the UPM190EP8 parent isolate clustered together with its bioreactor propagated progeny UPM190EP8BGMP1BP1 as distinct from the UPM190BGMP15 and its UPM190EP12BGMP15BP1 progeny, UPM0081EP8 and its UPM0081EP8BGMP1BP1, and UPM0081BGMP15 and its UPM0081BGMP15BP1 progeny (Figure 6).

### 3.3. Indirect Immunofluorescence Test

**Figure 7:**
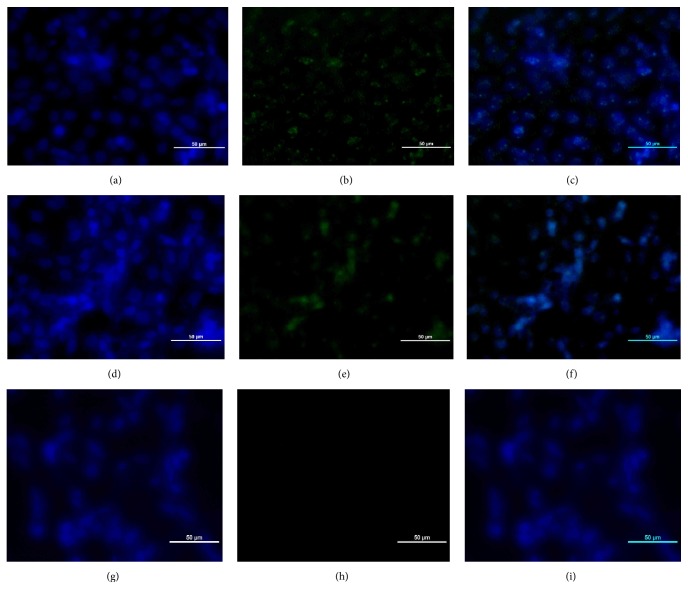
*The indirect immunofluorescence test on the bioreactor propagated UPM0081EP12BGMP15BP1 and UPM190EP12BGMP15BP1 showing high amount of IBDV antigen within the cytoplasm of BGM-70 cells at 72 hours after inoculation.* ((a) to (c)) BGM-70 cells infected with bioreactor propagated UPM0081EP12BGMP15BP1 and ((d) to (f)) UPM190EP12BGMP15BP1 showing positive signals for VP2 antigen (green) when stained with FITC-conjugated anti-chicken antibody raised against chicken anti-VP2 antibody. ((g) to (i)) Uninoculated control. Bar=50*μ*m.

**Figure 8:**
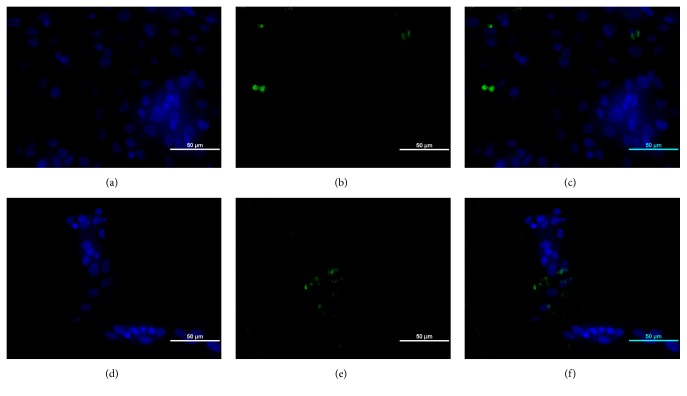
*The indirect immunofluorescence test on the flask propagated UPM0081BGMP15 and UPM190BGMP15 showing low amount of IBDV antigen within the cytoplasm of infected BGM-70 cells at 72 hours after inoculation.* ((a) to (c)) BGM-70 cells infected with flask propagated UPM0081BGMP15 and ((d) to (f)) UPM190BGMP15 showing positive signals for VP2 antigen (green) when stained with FITC-conjugated anti-chicken antibody raised against chicken anti-VP2 antibody. Bar=50 *μ*m.

The propagated IBDV viruses grew well in bioreactor due to the presence of expected positive signal seen as a green fluorescence within the cytoplasm of infected cells and showing high amount of viral antigen (Figure 7) compared to the isolates grown in tissue culture flasks (Figure 8).

## 4. Discussion

The main traditional means of producing vvIBDV seed virus for vaccine production has been using CEE from SPF chicken flocks [24]. This method is cumbersome and requires large facilities that are expensive and with limited scalability compared to animal cell cultures that can be propagated in large scale bioreactors for massive vaccine production [23]. Many continuous cell lines were evaluated for their ability to grow in different bioreactor platforms either as anchorage dependent or as suspension cultures with varying degree of successes [8, 23, 30, 31]. However, there is limited information on the growth of vvIBDV in bioreactor using a mammalian cell line, including BGM-70. Baby grivet monkey 70 cells have been well studied and characterized with respect to its ability to support the isolation and growth of many viruses including IBDV [14], but so far, no work was conducted to evaluate the cell line for its ability to support vvIBDV propagation in a bioreactor system.

The present study demonstrated the successful growth of vvIBDV in BGM-70 cell line on cytodex 1 microcarriers using stirring parameter of 12 rpm/5 minutes for the first 24 hours followed by 14 rpm/5 minutes for the rest of the culture period until harvest. At 37°C, 7.2 pH, and 5% C0_2_ vital culture conditions used in this study, the cells began attaching to the microcarriers as early as six hours ps and became confluent within 24 hours ps with cell density of 2.1 × 10^6^ cells/ mL. This was closely similar to the reports of Hundt* et al*. [32] who obtained a cell density of 1.2 × 10^6^ cells/mL in their study using adherent embryonic feline lung fibroblasts cells in a stirred tank bioreactor. The homogenous growth of BGM-70 cells on cytodex indicated that the culture conditions used in the present study were optimal for the cells using this propagation system. This demonstrated that the microcarrier-stirred tank bioreactor system used resulted in an increase in the volumetric cell yield as a result of its enhanced surface to volume ratio [32].

The virus yields obtained at 72 hours pi were 10^8.5^ TCID_50_/ mL and 10^8.7^ TCID_50_/ mL for UPM0081EP12BGMP15BP1 and UPM190EP12BGMP15BP1, respectively. In comparison to flask cultures (10^9.2^ TCID_50_/mL for UPM0081 and 10^9.5^ TCID_50_/mL for UPM190 isolates), the yield obtained in bioreactor was lower but the large volume of infectious virus obtained per batch of 2 L bioreactor per passage far outweigh the 5 mL obtained in a flask which will require 400 flasks to get the same 2 L volume. This is comparable to the virus yield obtained per 1 batch of 10 L wave bioreactor that was estimated to replace 1250 roller bottles with 0.25 L capacity needed to give the same volume [32] or to the 10^7.7^ TCID_50_ obtained for influenza virus within 20 hours pi [33]. This is an improvement in both infectious virus yield and biofactory technology. It seems that there is a better virus-cell interaction at the time of infection in a bioreactor system because of the increased agitation rate that is provided by the continuous rocking motion allowing better virus adsorption to the appropriate receptor for successful entry into the cell. This is similar to the periodic gentle shaking of flask done in a static flask culture during virus adsorption time before the addition of maintenance media [14]. It has been suggested that cell viability and survivability may be enhanced in bioreactor systems due to effective control of optimal culture conditions which may result in the increase in the virus replication phase within infected cells [32].

The limitation of this study is that the data detailing cell culture metabolism such as lactate and glucose uptake, glutamine, ammonium, glutamate, pyruvate, and other inhibiting metabolites and their accumulation were not studied; however, the advantages of the bioreactor system as means of propagating IBDV in comparison to the static flask culture were highlighted. This includes low costs (cytodex 1 microcarrier can be autoclaved and reused many times), ease of scalability, simple to use, adequate control of optimal culture conditions, and decreased microbial contamination. Thus, this bioprocessing system proves to be a favorable candidate for the scalability required in the manufacturing of viral vaccines for industrial application.

## Data Availability

The data used to support the findings of this study are available from the corresponding author upon request.
